# 1443. Characteristics of nursing homes with higher rates of invasive methicillin-resistant *Staphylococcus aureus* infections

**DOI:** 10.1093/ofid/ofad500.1280

**Published:** 2023-11-27

**Authors:** Isaac See, Kelly A Jackson, Rongxia Li, Prabasaj Paul, Kelly M Hatfield, Joelle Nadle, Susan Petit, Daniel Wurm, Allison Pall, Susan M Ray, Lee Harrison, Laura Jeffrey, Carmen Bernu, Ruth Lynfield, Ghinwa Dumyati, Anita Gellert, Tiffanie M Markus, William Schaffner, Kara M Jacobs Slifka, Nimalie D Stone

**Affiliations:** CDC, Atlanta, Georgia; U.S. Centers for Disease Control and Prevention, Atlanta, Georgia; CDC, Atlanta, Georgia; CDC, Atlanta, Georgia; Centers for Disease Control and Prevention, Atlanta, Georgia; California Emerging Infections Program, Oakland, California; Connecticut Department of Public Health, Hartford, Connecticut; Connecticut Department of Public Health, Hartford, Connecticut; Emory School of Medicine, Atlanta, Georgia; Emory University School of Medicine, Atlanta, Georgia; University of Pittsburgh, Pittsburgh, Pennsylvania; Johns Hopkins University, Baltimore, Maryland; Minnesota Department of Health, St. Paul, Minnesota; Minnesota Department of Health, St. Paul, Minnesota; New York Emerging Infections Program and University of Rochester Medical Center, Rochester, New York; University of Rochester Medical Center, Rochester, New York; Vanderbilt University Medical Center, Nashville, Tennessee; Vanderbilt University Medical Center, Nashville, Tennessee; Centers for Disease Control and Prevention, Atlanta, Georgia; CDC, Atlanta, Georgia

## Abstract

**Background:**

Invasive methicillin-resistant *Staphylococcus aureus* (MRSA) infections in nursing home residents now outnumber hospital-onset invasive MRSA infections. Elucidating characteristics of nursing homes with higher invasive MRSA rates could inform strategies to reduce rates of infections and prioritize facilities for prevention efforts.

**Methods:**

Using active laboratory- and population-based surveillance data, invasive MRSA cases (MRSA in a normally sterile body site in a surveillance area resident) were identified in 25 counties in 7 states in 2011-2015. Nursing home-onset cases were those in a patient residing in a nursing home 3 days before collection of the index MRSA specimen. Facility-level characteristics were obtained from Centers for Medicare & Medicaid Services datasets. Facilities with invasive MRSA rates (cases/100,000 resident-days) in the top 15^th^ percentile were defined as having “high rates” and a logistic regression model was used to assess factors associated with having high rates.

**Results:**

There were 626 total nursing homes included, with 2,772 invasive MRSA infections over the 5-year time period; 82% of the facilities had a least one invasive MRSA infection. The median invasive MRSA rate was 1.78 cases per 100,000 resident-days (interquartile range: 0.73-3.14), and nursing homes categorized as having high rates had rates of at least 4.04. In multivariable regression, documented presence of a resident with a multidrug-resistant organism, higher hospital-onset MRSA rates in the surveillance area, and greater proportions of residents who were male, on dialysis, in the facility < 100 days, or needed extensive assistance for bed repositioning were associated with having high invasive MRSA rates (Table). Increasing staffing of registered nurses (hours/resident/day) and a greater proportion of residents who were White were associated with lower rates.

Table

**Figure:**
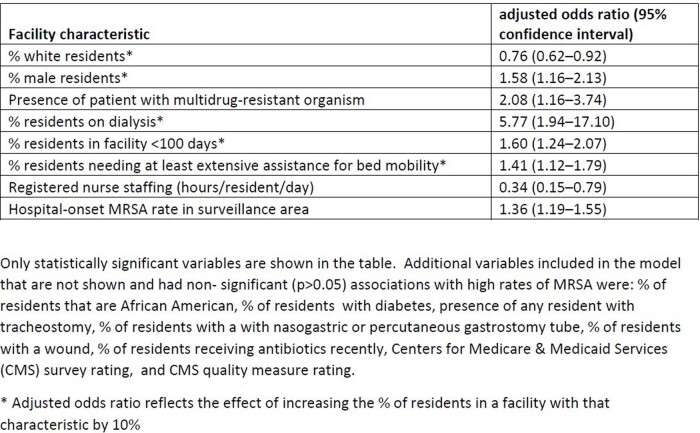

Adjusted odds ratios for facility-level factors associated with high rates (i.e., the top 15% of nursing homes) of nursing home-onset invasive methicillin-resistant Staphylococcus aureus (MRSA)

**Conclusion:**

Our findings suggest facilities with higher invasive MRSA rates are likely to serve residents with more clinical and functional care needs. Increasing hours of registered trained nurses might assist with reduction of invasive MRSA rates. Understanding why differences in rates are associated with the racial composition of residents may reveal health equity issues contributing to risk.

**Disclosures:**

**Lee Harrison, MD**, GSK: Advisor/Consultant|Merck: Advisor/Consultant|Pfizer: Advisor/Consultant|Sanofi: Advisor/Consultant **Ghinwa Dumyati, MD**, Pfizer: Grant/Research Support

